# Effect of recombinant human erythropoietin on postoperative anemia in children and adolescents undergoing osteosarcoma and potential influencing factors: a single-center retrospective study

**DOI:** 10.3389/fphar.2025.1637441

**Published:** 2025-11-05

**Authors:** Lulu Liu, Meili Lin, Wenjun Yang, Difei Yao, Huan Luo, Lingyan Yu, Haibin Dai

**Affiliations:** Department of Pharmacy, Second Affiliated Hospital, Zhejiang University School of Medicine, Hangzhou, China

**Keywords:** osteosarcoma, recombinant human erythropoietin, anemia, hemoglobin, rhEPO, perioperative period

## Abstract

**Background:**

Recombinant human erythropoietin (rhEPO) is an active glycoprotein secreted by the kidneys that improves anemia. The therapeutic role of rhEPO in anemia induced by surgical treatment after neoadjuvant chemotherapy in children and adolescents with osteosarcoma remains unclear. Additionally, factors influencing rhEPO efficacy in this context remain incompletely understood.

**Objective:**

This study aimed to assess the efficacy of rhEPO in treating postoperative anemia in children and adolescents with osteosarcoma and identify the key factors that may influence the therapeutic outcomes of this patient population.

**Methods:**

This retrospective study was conducted by pharmacists at a 4200-bed tertiary hospital in China, utilizing data extracted from the Hospital Information System. The study included patients aged 20 years or younger who were diagnosed with osteosarcoma and who underwent surgical treatment at the Second Affiliated Hospital, Zhejiang University School of Medicine, following the completion of two cycles of neoadjuvant chemotherapy. Data were collected between 1 January 2014, and 31 December 2023. Patients were divided into two groups on the basis of whether they received rhEPO treatment perioperatively: a control group (n = 36) and a treatment group (n = 68). The outcome data from both groups were compared to evaluate the effectiveness of rhEPO treatment and to identify factors affecting outcomes in patients with postoperative anemia.

**Results:**

This study included 64 males and 40 females (1.6:1 ratio), with a median age of 13 years and 2 months. Baseline anemia was present in 92.31% of patients (mild: 56, moderate: 40). Multiple linear regression analysis revealed that the use of rhEPO was significantly positively associated with length of hospital stay (B = 3.459, SE = 0.200, P = 0.005). Specifically, this result indicates that patients who received EPO had a mean length of hospital stay that was 3.459 days longer than that of patients who did not receive EPO. Univariate linear regression demonstrated that preoperative medication use independently predicted reduced hemoglobin concentrations on postoperative day 3 (OR = 6.75, 95% CI: 2.314 to 19.692, P < 0.001). Notably, Pearson correlation coefficients revealed a significant inverse relationship between total perioperative medication exposure and postoperative day 3 hemoglobin levels (r = −0.309, p = 0.007). Binary logistic regression analysis further indicated that preoperative medication use was independent predictors of outcomes in patients with postoperative anemia following osteosarcoma surgery.

**Conclusion:**

For hospitalized children and adolescents suffering from postoperative anemia following osteosarcoma surgery, rhEPO treatment can significantly shorten their hospital stay. Preoperative administration of rhEPO may be a key factor in rapidly helping patients to survive the postoperative hemoglobin trough. Given the inherent limitations of retrospective studies, further prospective research is warranted to validate these findings.

## 1 Introduction

Osteosarcoma is the most common malignant bone tumor. It predominantly affects adolescents between 10 and 30 years of age, with 10%–15% of these individuals being diagnosed with metastasis. Survival rates drop from 60% for localized cases to 20% for metastatic or recurrent cases (Meltzer and Helman, 2021; Su et al., 2024). Current treatment combines neoadjuvant chemotherapy, surgery, and adjuvant chemotherapy. This combination yields a 5-year survival rate of 60%–70% (Papakonstantinou et al., 2020; Smrke et al., 2021; Panez-Toro et al., 2023). First-line agents such as adriamycin, cisplatin, high-dose methotrexate, and ifosfamide often induce bone marrow suppression, leading to anemia in 70.69% of patients (Bajpai et al., 2017; Sanguanboonyaphong et al., 2022). Postoperative anemia is further exacerbated by surgical blood loss (Abu-Zaid et al., 2021; Zhou et al., 2023), necessitating hemoglobin (Hb) levels ≥8 g/dL to reduce the risk of mortality (Musallam et al., 2011; Cata, 2015; Moncur et al., 2021; Shander et al., 2023; Aoyama et al., 2024).

Anemia management involves allogeneic transfusion and pharmacotherapy. Risks associated with transfusion include infections, hemolytic reactions, and supply shortages (Kramer et al., 2017; Zhang et al., 2024). However, pharmacological approaches focus on erythropoiesis-stimulating agents (Roy et al., 2024), such as rhEPO, which stimulate red blood cell production. Studies have indicated that rhEPO effectively maintains Hb levels during chemotherapy, reduces transfusion needs, and improves perioperative anemia (Cao et al., 2020; Guan et al., 2020; Kaufner et al., 2020). In orthopedic surgeries (for instance, spinal fusion and hip/knee arthroplasty), preoperative rhEPO with iron supplementation optimizes Hb levels, lowers transfusion rates, and enhances recovery without increasing thrombosis risk (Cao et al., 2020; 2023).

However, evidence for the use of rhEPO in osteosarcoma surgery remains limited. Key uncertainties include its efficacy in minimizing postoperative Hb decline, optimal dosing timing. To address this, we retrospectively analyzed children and young patients with osteosarcoma treated at our institution, evaluated the ability of rhEPO to improve postoperative anemia and identified Hb-influencing factors. Our findings aim to guide clinical strategies for improving outcomes in this high-risk population.

## 2 Methods

### 2.1 Study design and population

This retrospective study was conducted by pharmacists at a 4200-bed tertiary hospital in China, utilizing data extracted from the Hospital Information System. The clinical data of patients diagnosed with osteosarcoma from 1 January 2014, to 31 December 2023, were retrospectively analyzed.

Among the 110 patients, 104 were selected according to the following criteria. The inclusion criterion was all osteosarcoma patients up to 20 years of age who underwent osteosarcoma surgery after completing 2 cycles of neoadjuvant chemotherapy. The exclusion criteria were as follows: (1) patients who underwent multiple surgeries and chemotherapy; (2) patients who did not complete the full course of treatment; (3) patients with organ failure or in a state of severe disease; (4) patients with a significant gap between chemotherapy and surgery (more than 2 weeks); (5) patients with incomplete or partially missing data; (6) patients with other comorbidities that may affect anemia, including chronic kidney disease, liver cirrhosis, severe heart disease, and severe hematological disorders; (7) patients taking preoperative medications such as aspirin that affect coagulation function; and (8) patients with obviously abnormal preoperative coagulation data from six tests.

**FIGURE 1:**
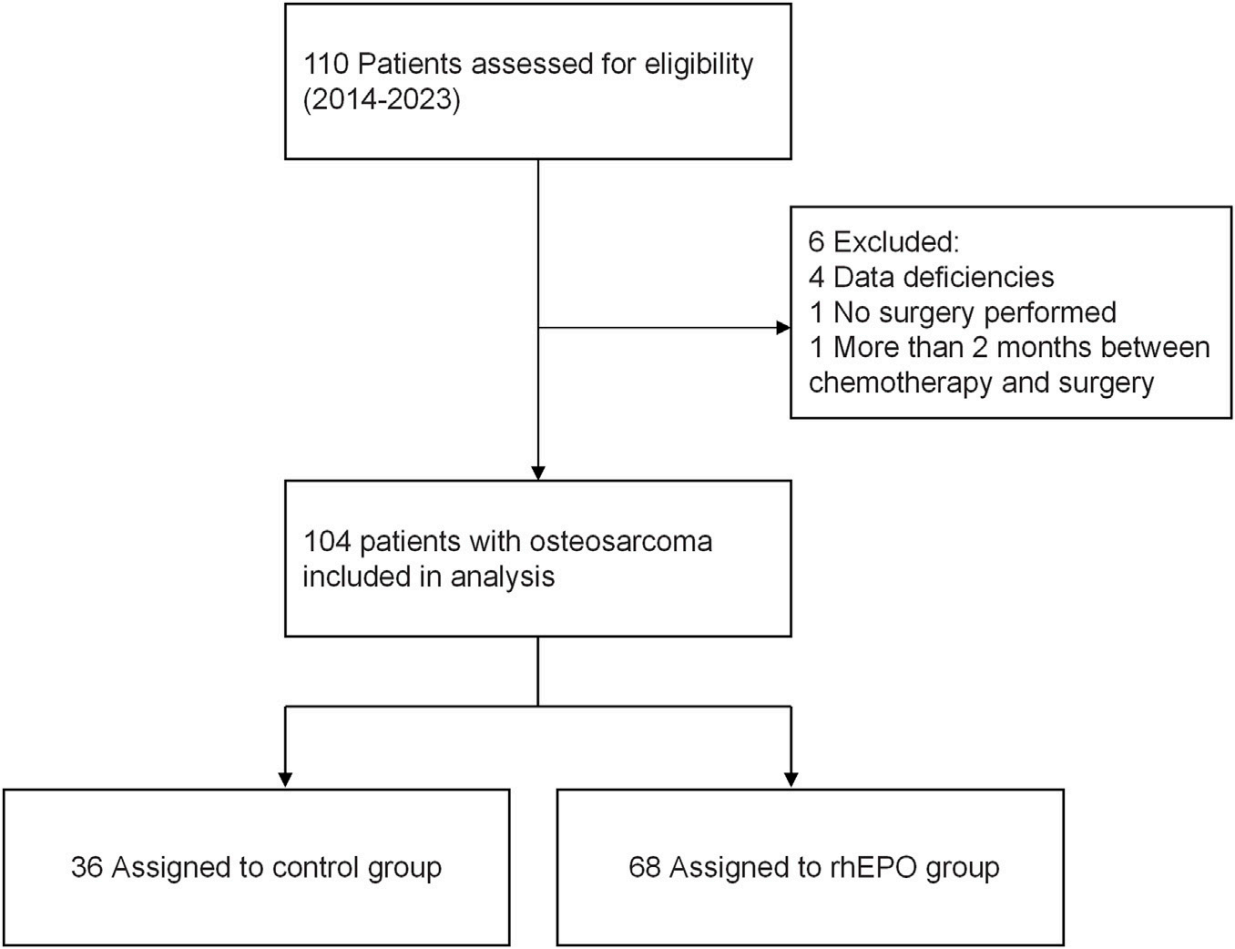
Trial flow chart for inclusion of patients with osteosarcoma.

Based on the baseline criteria, the 104 young patients were divided into control and rhEPO groups, depending on whether rhEPO was used during the perioperative period. Specifically, rhEPO was routinely indicated for patients undergoing osteosarcoma surgery with preoperative hemoglobin levels between 100 and 130 g/L. These patients were then assigned to two groups, comprising 36 and 68 patients, respectively (Figure 1). We compared sex, age, height, weight, comorbidities (the current patients had no history of hypertension, diabetes mellitus, heart disease, coronary heart disease, cerebrovascular accident, or renal disease, except for one history of asthma in the control group and two histories of asthma in the rhEPO group), intraoperative blood loss, or intraoperative blood transfusion between the two groups, as well as postoperative blood transfusion requirements, perioperative iron supplementation, and nutritional support.

After admission, patients in the medication group received rhEPO during the perioperative period, which was administered either once daily or every other day. The dosage was calculated on the basis of the patient’s body mass. All patients were treated with rhEPO (Shenyang Sansheng Pharmaceutical Co., Ltd., batch number 10,000 IU/bottle) for durations ranging from 1–18 days, with a mean treatment duration of 9.1 days. The osteosarcoma surgeries were performed by the same surgical team, and patients in both groups received conventional postoperative care following surgery.

In the rhEPO treatment group, a subset of patients received concurrent iron supplementation. The standard iron intervention protocol was an intravenous dose of 200 mg per administration, given three times weekly. The duration of iron supplementation was tailored to each patient’s clinical conditions, including serum ferritin levels, transferrin saturation, and hemoglobin recovery trends. Among the rhEPO group, 12 patients received postoperative nutritional support. The nutritional support was provided via enteral nutrition powder: 27.9 g per dose, dissolved in 125 mL of warm sterile water, and administered orally three times daily. The duration of nutritional support was adjusted based on clinical evaluation indicators, such as gastrointestinal function recovery, body weight stability, and serum albumin levels.

In addition, the criteria for postoperative blood transfusion were as follows: patients with hemoglobin levels less than 8 g/dL were eligible for blood transfusion. Additionally, patients with hemoglobin levels above 8 g/dL but presenting with significant symptoms of ischemia and hypoxia, such as anxiety, shortness of breath, or chest tightness, were also considered for transfusion.

The primary criterion for discharge was the stabilization of vital signs, with no complications or comorbidities that required further inpatient management. For instance, postoperative complications such as wound infection, wound discharge, or deep vein thrombosis would necessitate continued hospitalization. The final decision to discharge a patient was made by the attending physician, who conducted a comprehensive evaluation based on the patient’s specific condition.

### 2.2 Data collection, definitions and outcomes

Demographic and clinical data of each patient were collected, including sex, age, height, weight comorbidities (such as hypertension, diabetes, nephritis, heart disease, coronary heart disease, cerebrovascular accident), intraoperative blood loss, intraoperative blood transfusion, postoperative blood transfusion requirements, dose of rhEPO, frequency of use, perioperative iron supplementation, nutritional support, laboratory hemoglobin data, blood pressure monitoring, cardiovascular adverse events and thrombus-related events using ultrasound findings of venous thrombosis in both lower extremities.

The therapeutic effect of the medication was observed, with hemoglobin levels and changes used as the evaluation criteria. Given that the third postoperative day typically represents the nadir of hemoglobin levels during perioperative treatment, with subsequent improvement, the hemoglobin value on the third postoperative day was used as the evaluation index (Cao et al., 2020; 2023). Patients were considered to have achieved the standard if their hemoglobin level was ≥8 g/dL and if their clinical symptoms of anemia had improved (Cata, 2015; Chaudhry et al., 2022; Park et al., 2024).

The primary aim of this study was to evaluate the effect of rhEPO on the length of hospital stay. Secondary observations included Hb recovery on the third postoperative day and analysis of factors affecting its efficacy.

### 2.3 Statistical analysis

SPSS 27.0 software was used for statistical analysis. Continuous data are expressed as the means ± SDs, and categorical data are expressed as frequencies. Count data are expressed as the number of cases (n) and percentage (%). Multiple linear regression analysis were used to compare the length of hospitalization between the two groups of data. Univariate analysis of factors influencing the perioperative rhEPO outcome was performed via a chi-square test, and correlation analysis was used to assess the correlation between the baseline patient characteristics and postoperative anemia. The influencing factors were analyzed via a binary logistic regression model, and the difference was considered statistically significant if P < 0.05. We assigned values to the logistic analysis variables for improving postoperative anemia in Table 4.

## 3 Results

### 3.1 Patient characteristics

On the basis of the clinical data we collected, there was no significant difference in the baseline demographic characteristics of the 104 included patients between the two groups (P > 0.05) (Table 1). As shown in Table 1, with respect to comorbidities, a history of asthma was found in 1 patient in the control group and 2 patients in the rhEPO group, and no other comorbidities were found in these patients. Hemoglobin at admission was not significantly different between the two groups of patients.

**TABLE 1:** Clinical characteristics of the enrolled patients.

Factors	Control	EPO	P value
Mean age^a^ (years)	12.86 ± 2.46	13.41 ± 3.29	0.42
Gender, male/female	22/14	41/27	0.97
Height^a^ (cm)	153.88 ± 18.59	157.76 ± 17.30	0.30
Weight^a^ (kg)	44.72 ± 14.47	46.24 ± 14.90	0.63
Hb at admission^a^ (g/L)	98.30 ± 15.91	94.97 ± 14.75	0.30
Intraoperative blood loss^a^ (mL)	446.97 ± 320.87	546.62 + 660.89	0.767
Intraoperative blood transfusion^a^ (mL)	447.22 ± 425.26	487.94 ± 461.24	0.613
Postoperative blood transfusion, n (%)	4 (11.11%)	7 (10.29%)	0.898
Comorbidity, n (%)			
Hypertension	0	0	
Diabetes	0	0	
Ephritis	0	0	
Coronary heart disease	0	0	
Stroke	0	0	
History of asthma	1 (2.78%)	2 (2.94%)	0.56
Combination of relevant medications			
Iron supplements, n (%)	0	34 (50.00%)	<0.001
Nutritional Support, n (%)	0	12 (17.65%)	<0.001
Adverse events			
Postoperative thrombosis-related risk events, n (%)	0	0	
Hypertension, n (%)	1 (2.78%)	2 (2.94%)	0.462
Cardiovascular problems, n (%)	0	0	

^a^ The values are given as the mean and the standard deviation. Categorical data were examined using Pearson’s chi-square (χ^2^) test, continuous variables were analyzed using either the independent sample t-test or the Mann-Whitney U test, depending on the distribution of the data.

### 3.2 Clinical outcomes

#### 3.2.1 Hospital stays and thrombosis-related risk events

As illustrated in Table 1, a significant difference was observed in the combined use of iron supplements and nutritional support between the control group and the rhEPO group. However, no significant difference was found in postoperative blood transfusion between the two groups. As shown in Table 2, the association between rhEPO intervention and hospitalization duration was systematically evaluated through multiple linear regression analysis.

**TABLE 2:** Results of multivariate linear regression analysis on length of stayar regression.

Variables	B	SE	β	t Value	P value	VIF
Gender	1.400	1.121	0.119	1.249	0.215	1.230
Age (years)	0.315	0.308	0.171	1.022	0.310	3.814
Height (cm)	0.007	0.059	0.023	0.122	0.903	4.797
Weight (kg)	−0.097	0.057	−0.253	−1.686	0.095	3.075
Hb at admission (g/L)	0.024	0.040	0.062	0.594	0.554	1.504
Intraoperative blood loss	0.001	0.002	0.095	0.603	0.548	3.397
Intraoperative blood transfusion	0.002	0.002	0.221	1.342	0.183	3.682
Use of rhEPO	3.459	1.190	0.288	2.906	0.005	1.340
Iron supplements	−1.426	1.272	−0.112	−1.121	0.265	1.360
Nutritional Support	−3.367	1.721	−0.188	−1.956	0.054	1.264
Postoperative blood transfusion	2.302	1.685	0.124	1.366	0.175	1.123

*R* ^2^ = 0.325, adjusted value *R* ^2^ = 0.245. VIF, stands for variance inflation factor. The VIF, for each independent variable is < 5, indicating that the multicollinearity among variables is negligible. B and SE, represent the unstandardized coefficient βand the standard error, respectively. Taking length of hospital stay as the dependent variable and use of EPO, as the independent variable, while adjusting for age, sex, height, weight, admission hemoglobin level, intraoperative blood loss and transfusion volume, iron supplement use, and postoperative nutritional and transfusion support. *R* ^2^ = 0.325, adjusted value *R* ^2^ = 0.245.

In this study, the length of hospital stay was designated as the dependent variable, with the use of rhEPO being the primary independent variable. Additionally, the model was adjusted for a range of potential confounding factors, including age, gender, height, weight, hemoglobin level at admission, intraoperative blood loss, intraoperative blood transfusion volume, iron supplementation, postoperative nutritional support, and postoperative blood transfusion. Multiple linear regression analysis revealed that the use of rhEPO was significantly positively associated with the length of hospital stay (B = 3.459, SE = 0.200, P = 0.005). Specifically, this result indicates that patients who received rhEPO had a mean length of hospital stay that was 3.459 days longer than that of patients who did not receive rhEPO. Other factors did not have a significant impact on the duration of the hospital stay.

Among these patients, no postoperative thrombosis-related risk events were observed via bilateral limb deep vein ultrasound, and no cardiovascular adverse events were reported. Elevated blood pressure was observed in only 2 cases in the rhEPO group and 1 case in the control group, with no statistically significant difference between the two groups (Table 1).

#### 3.2.2 Factors influencing the efficacy of anemia treatment

**FIGURE 2:**
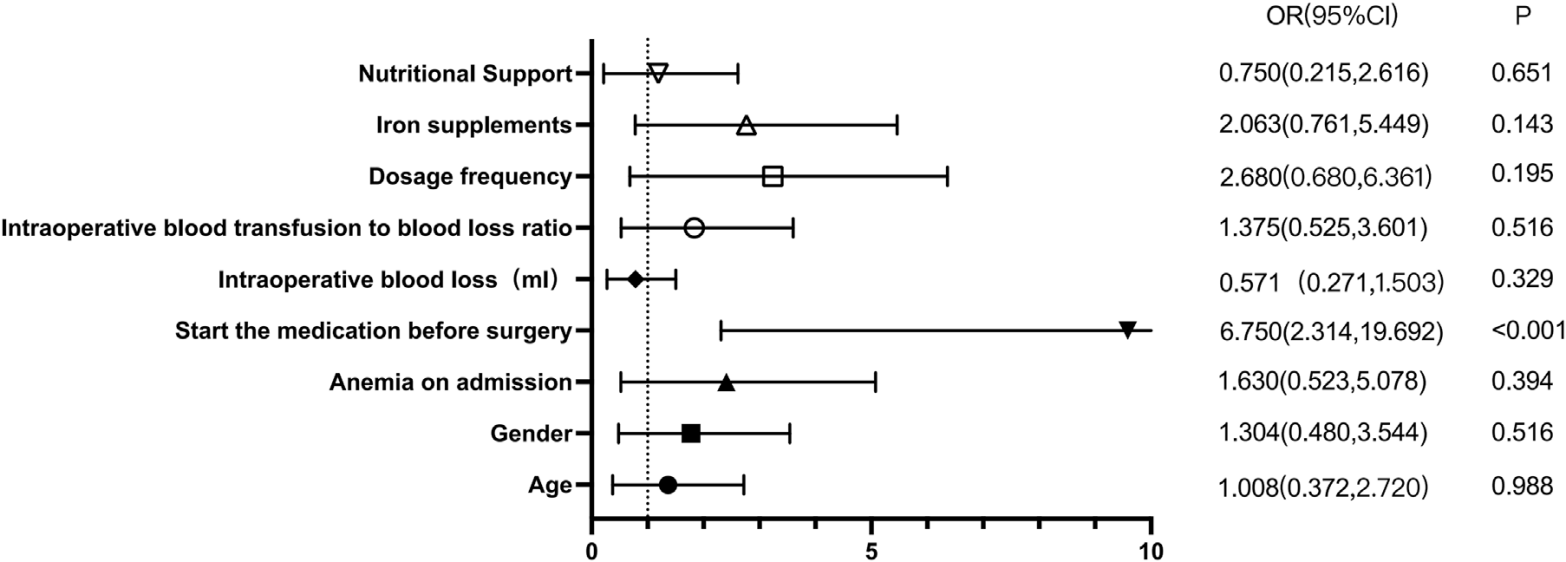
rhEPO univariate analysis of perioperative treatment outcomes.

We set a hemoglobin level ≥8 g/dL on the third postoperative day to achieve a therapeutic effect. Among all patients, 38 patients (55.88%) met the criteria, whereas 30 (44.12%) did not. One-way analysis of variance was performed to assess the relationships between patient age, sex, anemia status at admission, preoperative drug administration, intraoperative blood loss, intraoperative blood transfusion, and blood loss/transfusion ratios and postoperative hemoglobin levels on the third postoperative day. In the unifactorial analysis of the therapeutic effect in patients with osteosarcoma with postoperative anemia, only preoperative drug administration was significantly different (OR = 6.75, 95% CI: 2.314 to 19.692, p < 0.001)), whereas the differences in the remaining variables were not statistically significant (P > 0.05; Table 3; Figure 2).

**TABLE 3:** rhEPO univariate analysis of perioperative treatment outcomes.

Influence factor	Fail to meet the standards^a^	Reach the standard^a^	χ2	P Value
Age				
6–14	19	24	0.000	0.988
15–20	11	14		
Gender				
Men	20	23	0.272	0.601
Women	10	15		
Anemia on admission				
Yes	24	27	0.716	0.394
No	6	11		
Start the medication before surgery				
Yes	8	27	13.223	<0.001
No	22	11		
Intraoperative blood loss (ml)				
<500	14	23	1.298	0.329
≥500	16	15		
Intraoperative blood transfusion to blood loss ratio				
≥1	15	16	0.421	0.516
<1	15	22		
Dosage frequency				
qd	24	25	1.681	0.195
qod	6	13		
Iron supplements				
Yes	12	22	2.147	0.143
No	18	16		
Nutritional support				
Yes	6	6	0.205	0.651
No	24	32		

^a^ We set a hemoglobin level of ≥8 g/dL on the third postoperative day as the therapeutic target. A level meeting this standard indicates therapeutic efficacy, while a level below 8 g/dL indicates failure to meet the standard.

**FIGURE 3:**
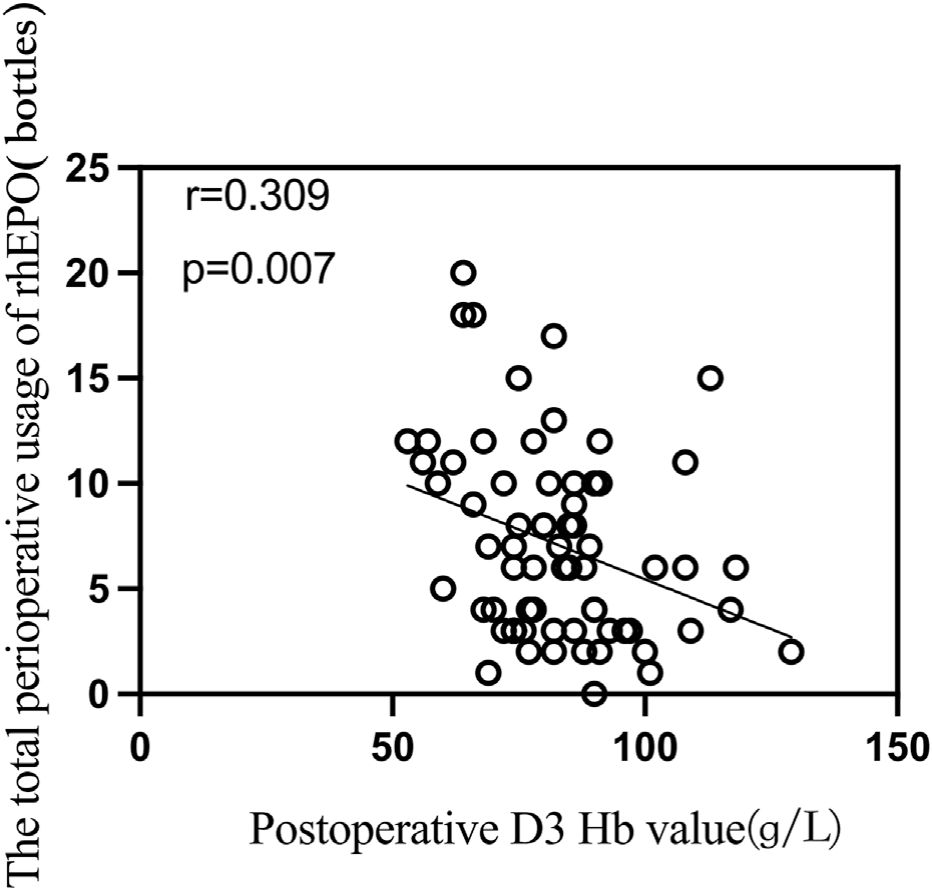
Correlation analysis between total perioperative usage of rhEPO and postoperative day 3 hemoglobin levels.

We analyzed the correlations among the patient’s height, weight, body mass index, hemoglobin level at admission, red blood cell count, hematocrit, intraoperative blood loss, total perioperative medication dosage (units), and hemoglobin levels on the first and third days after surgery. The results revealed a negative correlation between the total amount of rhEPO used and the hemoglobin level on the third postoperative day (r = −0.309, p = 0.007). The results are presented in Figure. 3.

In the binary logistic regression analysis of the treatment effects on patients with osteosarcoma with postoperative anemia, the variables that were found to be statistically significant in the univariate analysis were used as independent variables, whereas the classification of patients’ postoperative treatment effects served as the dependent variable in the binary logistic analysis. The results showed that preoperative administration of rhEPO had a significant effect on hemoglobin values on the third postoperative day. Tables 4, 5 present the methods of variable assignment and the results of the binary logistic regression analysis.

**TABLE 4:** Logistic analysis of variable assignment for perioperative treatment outcomes in patients with osteosarcoma.

Variant	Assignment	
Implicit variabl	Therapeutic effects	0 = reach the standard; 1 = fail to meet the standards
Independent varia	start the medication before surgery	0 = Yes 1 = no
	Total perioperative use (bottle)	Continuous variable

**TABLE 5:** Results of a binary logistic analysis of perioperative patient outcomes.

Influence factor	β	SE	Wald-χ2	OR	95%CI	*P* Value
Preoperative drug administration	2.205	0.607	13.316	9.070	2.670–28.230	<0.001
Total perioperative use	−0.135	0.344	3.797	0.512	0.765–0.998	0.051

## 4 Discussion

Patients with osteosarcoma often experience severe anemia in the postoperative period due to the dual effects of chemotherapy and surgery; this test is much more difficult than the average postoperative orthopedic procedure is and has a direct effect on the survival of patients with tumors (Cata, 2015; Makar et al., 2024). However, owing to the relative rarity of patients with osteosarcoma, relatively few clinical studies exist on this topic, and relatively few reports exist in the available literature on perioperative blood management protocols that specifically address this issue. Current perioperative blood management strategies generally focus on minimizing blood transfusions and optimizing patient outcomes through comprehensive management of anemia, blood loss, and coagulopathy. These strategies include (1) optimizing preoperative hemoglobin through a multidisciplinary approach; (2) minimizing perioperative blood loss; and (3) adhering to evidence-based transfusion guidelines (Holt et al., 2016).

Erythropoietin is an effective drug for the treatment of anemia. The mechanism of action of rhEPO involves stimulating hematopoietic stem cells in the bone marrow, thereby increasing the amounts of raw materials available for hemoglobin synthesis to promote the maturation and release of red blood cells into the bloodstream. In addition, rhEPO can activate the division of primitive red blood cells to promote the proliferation and release of reticulocytes, which ultimately leads to increased levels of hemoglobin, erythrocytes, and reticulocytes in the patient’s body to reduce the number of blood transfusions. Van Iperen C reported that rhEPO levels in the blood were significantly elevated after smajor surgery and peaked on the 5th postoperative day (Van Iperen et al., 1998). Moreover, in a mouse model of cancer, the hemoglobin concentration in the rhEPO-treated group was significantly greater than that in the control group. Currently, researchers can genetically intervene with erythropoietin at the molecular level and better express the gene locus for anemia treatment, suggesting that rhEPO is effective for both surgery and cancer-related anemia (Nguyen et al., 2013; Cao et al., 2020). Although rhEPO has been associated with venous thromboembolism, elevated blood pressure, and adverse cardiovascular events, previous studies have shown that rhEPO does not increase the risk of thrombosis following orthopedic surgery (Ruan et al., 2020). This study confirms that finding, with no significant increase in hypertension or cardiovascular event risk.

We chose a target population within the age of 20 years because the first peak incidence of osteosarcoma is within the age of 20 years, and the treatment strategy for this population is essentially the same (Ahlawat et al., 2024). In this study, rhEPO treatment effectively improved anemia. Specifically, the use of rhEPO significantly shortened hospital stays, with an average reduction of up to 3.459 days. These findings indicate that rhEPO can effectively prolong patients’ hospitalization and recovery periods while also lowering medical costs, possibly because patients need time to recover from chemotherapy after experiencing myelosuppression. The use of erythropoietin significantly reduces the waiting time before surgery or hospitalization after surgery because patients who were not treated previously needed to wait for hemoglobin recovery after chemotherapy, prolonging their hospitalization. Conversely, patients who had mild anemia before surgery and severe postoperative anemia due to blood loss during surgery needed to improve their anemia, thereby prolonging hospitalization.

The main cause of perioperative anemia in osteosarcoma patients is the insufficient erythropoietic function in the bone marrow, likely due to tumor consumption, surgical blood loss, and chemotherapy-induced suppression. rhEPO can improve anemia through several mechanisms. It binds to its receptor and activates the EPO-EPOR-JAK2-STAT5 signaling pathway, which promotes the proliferation and differentiation of erythroid progenitor cells and inhibits apoptosis, thus restoring erythrocyte production in suppressed bone marrow (Bunn, 2013). EPO also regulates iron metabolism by reducing ferritin levels and enhancing iron absorption and utilization, providing sufficient materials for erythropoiesis (Rishi and Subramaniam, 2017). Additionally, it has anti-inflammatory effects by suppressing cytokine production and alleviating inflammatory responses (Tsavlis et al., 2024). During chemotherapy, EPO can reduce chemotherapy drug toxicity (Miltenburg and Boogerd, 2014; Liu et al., 2024). These mechanisms collectively enhance erythropoiesis, alleviate anemia symptoms, and improve patients’ quality of life.

In previous studies, iron supplements combined with rhEPO were expected to improve postoperative recovery by addressing anemia. Iron provided essential materials for red blood cell production, while rhEPO stimulated this process (Correnti et al., 2022; Maltaneri et al., 2024), significantly enhancing erythropoietic efficiency. Enteral nutrition powder supported recovery by offering comprehensive nutrition and enhance hemoglobin levels, decrease the incidence of infections, and accelerate the recovery process (Liu et al., 2013; Martínez-Ortega et al., 2022). However, our study showed that while rhEPO significantly shortened hospital stays, the contributions of iron supplements and enteral nutrition powder were not statistically significant. Chemotherapy for osteosarcoma caused bone marrow suppression and chronic inflammation, leading to hepcidin-mediated iron sequestration (Rishi and Subramaniam, 2017). This reduced the effectiveness of iron supplements in supporting erythropoiesis. Additionally, chemotherapy damaged erythroid progenitor cells and decreased EPO receptor numbers, further suppressing rhEPO’s effects (Doshi et al., 2013). Consequently, iron supplementation did not significantly improve postoperative recovery or shorten hospital stays. Enteral nutrition powder was most beneficial for perioperative patients with moderate-to-severe malnutrition and impaired digestion/absorption, criteria that osteosarcoma patients may not meet, thus limiting the impact of nutritional support on length of stay.

In this study, no significant difference was observed between the rhEPO group and the untreated group in postoperative transfusion requirements. First, the small sample size limited the statistical power of the analysis, which may have masked rhEPO’s potential efficacy. Second, in the rhEPO group, some patients only started treatment postoperatively—with delayed administration and possibly insufficient dosing. This may have prevented the full erythropoietic effect from being realized, thereby limiting any reduction in transfusion requirements. Finally, postoperative transfusion requirements in osteosarcoma patients are affected by multiple factors, rhEPO was not a decisive factor (Dupuis et al., 2015).

Preoperative administration of rhEPO significantly elevates hemoglobin levels postoperative day 3, with this effect being particularly pronounced when comparing the preoperative administration group to the non-preoperative administration group. This can be attributed to two main factors. First, patients who have completed chemotherapy often experience myelosuppression, and the use of erythropoietin can help mitigate this condition. Second, erythropoietin mobilization requires 4–5 days; therefore, preoperative medication can promote the immediate mobilization of blood. This helps control the decrease in hemoglobin during the postoperative hemoglobin trough period, enabling patients to successfully navigate through this critical phase (Van Iperen et al., 1998). Previous studies (Cao et al., 2020) have indicated that a 3-day course of preoperative rhEPO combined with iron supplementation significantly reduces intraoperative bleeding and improves postoperative Hb levels without significantly increasing the risk of complications. Furthermore, preoperative rhEPO treatment may be a more effective blood conservation regimen for total knee arthroplasty (TKA) surgery. The results of this study align with those of previous studies, suggesting that the timing of the administration of preoperative medication is crucial since erythropoietin mobilization requires time.

We found a slight negative correlation between the total perioperative dose of rhEPO and postoperative hemoglobin values on postoperative day 3 in a correlation analysis, but this correlation was not significant in binary logistic regression modeling (Table 5). This may be due to the relationship between treatment duration and initial Hb levels. Lower Hb on day 3 may indicate a need for longer erythropoietin therapy to achieve the desired therapeutic goal, though the impact on outcomes remains uncertain. With a fixed daily dose of rhEPO, a higher total dose indicates a longer administration period. Patients with lower initial Hb levels require more time to reach the target Hb range of 10–13 g/dL. As indicated in the literature (Doshi et al., 2013), CKD patients on dialysis with a baseline Hb of 8 g/dL may need 10–15 weeks of treatment, while those with a baseline Hb near 10 g/dL may need only 6–8 weeks. This extended treatment period results in a higher total dose, creating the observed negative correlation. This correlation reflects the adjustment of treatment duration based on disease severity rather than a direct causation of Hb reduction by the rhEPO dose itself. The goal of treatment is to elevate Hb to the target range, and the initial Hb level determines the required treatment duration. Therefore, the negative correlation is a result of the treatment strategy rather than the effect of the drug dose on Hb levels.

Beyond the role in erythropoiesis, rhEPO have been reported to promote tumor cell growth or survival. A leading hypothesis proposed to explain these safety findings was that functional erythropoietin receptors (EPORs) are expressed in tumor cells and/or endothelial cells, allowing ESAs to directly stimulate tumor growth and/or counteract tumor-ablative therapies. A recent review evaluating evidence supporting the EPO–EPOR tumor-stimulation hypothesis highlighted critical limitations in much of the existing research (Elliott et al., 2006). Specifically, putative positive results were frequently confounded by two key issues: the lack of controls to rule out false-positive effects, and the use of nonspecific reagents. Additionally, EPOR expression levels outside the erythroid compartment—including on tumor cells—are extremely low. Current data fail to convincingly demonstrate that such low-level EPORs can bind sufficient amounts of ESAs to elicit a functional biological response (Sinclair et al., 2010; Swift et al., 2010). Collectively, the available evidence indicates that ESAs do not directly stimulate tumor cells. Furthermore, the cytoprotective and other nonhematopoietic effects of ESAs reported in previous studies do not appear to result from direct ESA-EPOR signaling in nonerythroid cells (Steve and Angus, 2012).

Our study has several limitations. First, it is single-center with a modest sample size, the generalizability of the findings remains limited. These limitations stem from the low incidence of this rare pediatric disease, as well as the practical challenges associated with collecting cases from other institutions. Nevertheless, our study offers valuable insights into osteosarcoma, the most common primary malignant bone tumor in this pediatric population. Second, the retrospective design of this study introduces potential confounding factors and selection bias. In addition to the use of rhEPO combined with iron supplements, perioperative nutritional support also positively impacts the improvement of postoperative anemia. However, these factors are intertwined, making it difficult to completely exclude them, which may introduce some interference into the study results. Therefore, a prospective, randomized, controlled trial is recommended to reduce bias and confounding factors. Third, hospitalization durations varied, preventing standard 1-week postoperative hemoglobin comparisons, Follow-up was limited to the third postoperative day. This was due to the varying patient recovery times, short hospital stays, and data gaps in our system. Obtaining informed consent for follow-up from all patients also took time. Previous studies have suggested that rhEPO has adverse effects on long-term cancer survival, including potential tumor progression. In our study, we did not conduct long-term follow-up of these patients to assess these risks (Pradeep et al., 2015). In future studies, rigorous protocols should be developed to precisely manage the timing of administration, dosage, dosage frequency, and total amount of medication. Such improvements may help identify the most effective timing and total dosage for treatment. These findings may also clarify the influence of rhEPO on long-term patient survival.

## 5 Conclusion

Our findings demonstrate that rhEPO significantly shortens the duration of hospital stays, with no significant increase in adverse events observed. Notably, preoperative administration of rhEPO emerges as a potential critical factor contributing to this favorable outcome. However, we also acknowledge the inherent limitations of the present study, and thus stress the need for further well-designed research to validate, refine, and extend these preliminary findings.

## Data Availability

The original contributions presented in the study are included in the article/supplementary material, further inquiries can be directed to the corresponding authors.
